# Assessment of white matter microstructure integrity in subacute postconcussive vestibular dysfunction using NODDI

**DOI:** 10.1162/imag_a_00147

**Published:** 2024-04-10

**Authors:** Joseph A. Behnke, Vishwadeep Ahluwalia, Jeremy L. Smith, Benjamin B. Risk, Jianna Lin, Russell K. Gore, Jason W. Allen

**Affiliations:** a Department of Radiology and Imaging Sciences, Emory University School of Medicine, Atlanta, GA, United States; b Georgia Institute of Technology, Atlanta, GA, United States; c GSU/GT Center for Advanced Brain Imaging, Atlanta, GA, United States; d Department of Biostatistics and Bioinformatics, Rollins School of Public Health, Emory University, Atlanta, GA, United States; e University of Central Florida College of Medicine, Orlando, FL, United States; f Shepherd Center, Atlanta, GA, United States; g Wallace H. Coulter Department of Biomedical Engineering, Georgia Institute of Technology and Emory University, Atlanta, GA, United States; h Department of Radiology & Imaging Sciences, Indiana University School of Medicine, Indianapolis, IN, United States

**Keywords:** concussion, traumatic brain injury, diffusion imaging, vestibular impairment

## Abstract

Vestibular symptoms, such as dizziness and balance impairment, are frequently reported following mild traumatic brain injury (mTBI) and are associated with a protracted recovery, yet the underlying neuroanatomical substrates remain unclear. The present study utilized advanced diffusion MRI (dMRI) techniques including both conventional diffusion tensor imaging (DTI) and neurite orientation dispersion and density imaging (NODDI) to investigate microstructural white matter integrity in individuals with postconcussive vestibular dysfunction (PCVD) within the subacute injury period (median of 35 days from injury; IQR of 23). Study participants included 23 individuals with subacute PCVD and 37 healthy control subjects who underwent imaging and comprehensive clinical vestibular testing. Between-group voxelwise analysis of differences in white matter revealed areas of higher intra-neurite volume fraction (V_In_) and isotropic volume fraction (V_Iso_) within PCVD subjects compared to controls, which involved overlapping regions within the left hemisphere of the brain. Affected areas of higher V_In_ and V_Iso_ included the superior longitudinal fasciculus (SLF) and superior and posterior corona radiata (SCR and PCR, respectively). We examined the relationship between clinical vestibular measures and diffusion metrics including DTI (fractional anisotropy [FA], mean diffusivity [MD], radial diffusivity [RD] and axial diffusivity [AD]) and NODDI (intraneurite volume fraction [V_In_], isotropic volume fraction [V_Iso_], dispersion anisotropy [DA], orientation dispersion index_Total/Primary/Secondary_ [ODI_T/P/S_]) within 32 regions-of-interest. Clinical vestibular measures included self-reported measures, including the Dizziness Handicap Inventory, Visual Vertigo Analog Scale, and Vestibular/Ocular-Motor Screening, as well as objective vestibular testing using the sensory organization test. Significant correlations were found with clinical measures across all diffusion maps (except DA), within various regions of interest (ROIs), including SLF, SCR, and PCR. These results implicate several important association bundles that may potentiate sensory processing dysfunction related to PCVD. Whether these neuroanatomical differences found within the subacute phase of PCVD are in response to injury or represent preexisting structural variations that increase vulnerability to sensory processing dysfunction is unclear and remains an active area of study.

## 1. INTRODUCTION

Mild traumatic brain injury, also referred to as concussion, accounts for approximately 80% of the estimated 3 million traumatic brain injuries each year in the US (Centers for Disease Control and Prevention, 2003, 2015). Although most patients who sustain a concussion recover within 10–14 days, 10–40% of affected individuals experience persistent post-concussion symptoms beyond 6 months following injury (Fordal et al., 2022; Voormolen et al., 2018), indicating that concussion may result in long-term neurobiological changes (King et al., 2019; Nelson et al., 2019). Vestibular impairment following concussion is frequently reported within civilian and military populations (Asken & Rabinovici, 2021; Barnes et al., 2018; Luethcke et al., 2011; Rosenfeld et al., 2013; Warden, 2006), affecting up to 80% of concussed patients (Hoffer et al., 2010; Maskell et al., 2006). Vestibular impairment is associated with protracted recovery (Anzalone et al., 2017), and delayed return to both athletic competition (Chorney et al., 2017) and combat (Kennedy et al., 2012). Despite these findings, there remains a limited understanding of the neuropathological changes underlying vestibular impairment following concussion.

Vestibular function involves multimodal processing, including integration of information from central nervous system regions related to visuospatial, somatosensory, and motor function (Indovina et al., 2020). Recent studies have begun to characterize structural connectivity of vestibular circuitry using diffusion MRI (dMRI) (Indovina et al., 2020; Kirsch et al., 2016; Wirth et al., 2018). In addition to its application within the healthy state, dMRI provides a non-invasive means to study structural integrity within the brain following concussion. In response to mechanical injury, white matter (WM) tracts undergo torsional and sheer stress (Johnson et al., 2013; McKee & Daneshvar, 2015), which ultimately disrupts water movement within various subcellular and extracellular compartments that is detectable using dMRI. Historically, white matter microstructure integrity has been modeled using diffusion tensor imaging (DTI) (Soares et al., 2013). Applications of DTI to concussion have revealed changes within WM microstructure, including decreased fractional anisotropy (FA) as early as 72 h following concussion, which is suggestive of axonal injury (Palacios et al., 2020; Zimmerman et al., 2021). At the same time, mean diffusivity (MD) is increased, which is believed to correspond to axonal swelling and inflammation (Mac Donald et al., 2011; Toth et al., 2013). Changes in DTI metrics can also be detected more than 6 months post-injury (Dean et al., 2015; Mac Donald et al., 2011) and are correlated with postconcussive symptom severity and recovery time (Alhilali et al., 2014; Dean et al., 2015). Even in absence of overt clinical signs and symptoms, emerging data suggest evidence of structural changes following sub-concussive injuries (Bahrami et al., 2016; Bazarian et al., 2012; Hirad et al., 2019; Maged et al., 2023). Recent attention has sought to understand the relationship between structural pathological changes and their respective clinical deficits following concussion, including changes in white matter related to memory/cognition (Chung et al., 2019; Muller et al., 2021; Wu et al., 2018), headache (Ghodadra et al., 2016), and vestibular dysfunction (Alhilali et al., 2014; Gard et al., 2022; Jang et al., 2021).

Despite its growing use, conventional DTI provides limited specificity in its characterization of white matter pathology (Zhang et al., 2012). For example, changes in FA may be attributed to a diverse array of pathologies related to axonal density, myelination, and extracellular/intracellular water shifts (O’Donnell & Westin, 2011). Furthermore, tensor fitting within DTI modeling assumes one fiber type per voxel, which poses a challenge when characterizing non-uniform areas of crossing fibers and multi-fiber composition with various orientations (Lanyon, 2012), such as that of the corona radiata and superior longitudinal fasciculus (O’Donnell & Westin, 2011; Timmers et al., 2016). A recently developed multi-shell diffusion signal modeling technique, known as neurite orientation dispersion and density imaging (NODDI) addresses these limitations associated with DTI. NODDI provides a biophysical representation of tissue microstructure that involves estimation of a three-compartment model corresponding to regions of (i) intraneurites, that is, axons and dendrites; (ii) extraneurites, that is, soma and glia; and (iii) free water, that is, cerebrospinal fluid (Zhang et al., 2012). Several recent reports using NODDI have revealed subtle pathologies related to concussion which are not detected using less-specific conventional DTI parameters (Muller et al., 2021; Palacios et al., 2020) and therefore provide a better representation of individual tissue microstructure within clinically acceptable acquisition time (Zhang et al., 2012). Implementation of more specific diffusion-based models will provide improved characterization of subtle neuropathological changes underlying clinical phenomenon, including those related to post-concussive sequelae.

The present study utilized a complementary approach combining DTI with NODDI diffusion metrics to assess WM microstructure integrity after conccusion in individuals with subacute postconcussive vestibular dysfunction (PCVD). The subacute phase of injury represents a clinically important time for a subset of individuals who subsequently develop persistent symptoms. The specific aims of this study sought to (1) identify whether structural differences exist between individuals with subacute PCVD and healthy controls, and (2) assess the potential relationship between neuroanatomical white matter regions and clinical vestibular dysfunction as measured using self-reported and objective measures. Together, our findings implicate several important white matter association tracts that may potentiate symptoms related to subacute PCVD.

## 2. METHODS

### 2.1. Ethics

This study was approved by our local Institutional Review Board at Emory University (IRB00105844). All subjects provided informed consent.

### 2.2. Study population

This prospective study included 37 healthy control subjects without a history of PCVD and 23 subacute PCVD patients. Inclusion criteria for subjects with PCVD were a diagnosis of concussion, as defined by the World Health Organization Collaborating Center for Neurotrauma Task Force (Carroll et al., 2004) and clinical evidence of vestibular impairment defined as a subjective report of dizziness and/or imbalance, clinical visual motion sensitivity symptoms, and provocation of symptoms during Vestibular/Ocular-Motor Screening (VOMS) (Mucha et al., 2014). Exclusion criteria for both control and PCVD subjects included a history of moderate or severe head injury, intracranial hemorrhage, seizure disorder, prior neurologic surgery, peripheral neuropathy, musculoskeletal injuries affecting gait and balance, and chronic drug or alcohol use. Additionally, subjects with abnormal head impulse testing findings or videonystagmography consistent with peripheral vestibular hypofunction or benign paroxysmal positional vertigo were excluded.

### 2.3. Self-reported clinical measures

Subject participants completed the Post-Concussion Symptom Scale (PCSS) (Lovell et al., 2006). Self-reported and subjective measures included the Dizziness Handicap Inventory (DHI) (Jacobson & Newman, 1990), Visual Vertigo Analog Scale (VVAS) (Dannenbaum et al., 2011), and VOMS (Mucha et al., 2014). Subjective affective clinical measures were also examined using the Becks Anxiety Inventory (Beck et al., 1988) and Becks Depression Inventory (Beck et al., 1996). Group-wise differences in self-reported clinical measure scores were assessed by Mann-Whitney U test with adjustment for multiple comparisons using Benjamini-Hochberg correction. In three subacute PCVD subjects, a single VOMs subscore was missing, resulting in a missing composite VOMS. Two healthy controls and four subacute PCVD subjects did not have VVAS or DHI data. Subjects with missing data for certain clinical measures were omitted and not included within linear regression analyses.

### 2.4. Sensory Organization Test (SOT)

The Sensory Organization Test (SOT) is an objective measure of dynamic posturography (Nashner & Peters, 1990) using an immersive virtual projection system (Bertec FIT CDP/IVR, Bertec Immersive Labs, Columbus, Ohio) equipped with dual-balance force plates. SOT has established validity for asessing vestibular impairment after stroke (Bonan et al., 2004), traumatic brain injury (Guskiewicz et al., 2001; Ruhe et al., 2014), and vestibular disorders (Whitney et al., 2006). The SOT comprised six sensory conditions that test a participant’s ability to maintain equilibrium, operationalized by postural sway and center of pressure, over three 20-s trials per condition. Conditions include the following: (1) eyes open while on a fixed support surface and facing a static immersive scene; (2) eyes closed on a fixed surface; (3) eyes open with sway-referenced visual surround (i.e., the visual scene changes synchronously with variations in center of gravity); (4) eyes open on a sway-referenced support surface (i.e., the support surface changes synchronously with variations in center of gravity); (5) eyes closed on a sway-referenced support surface; and (6) eyes open on both a sway-referenced support surface and visual surround. For each of the six conditions, an equilibrium score is generated from the center of gravity sway and an overall SOT composite score is reported representing performance on all six conditions, with higher scores representing better balance performance, that is, less body sway. In addition, standardized sensory analysis ratios/subscores are calculated representing utilization of specific sensory strategies, including visual, vestibular, and somatosensory strategies (Row et al., 2019). Lastly, the visual preference ratio corresponds to a subject’s reliance on visual information to maintain balance even with unreliable visual information (Perucca et al., 2021). Group-wise differences in SOT scores were assessed by Mann-Whitney U test with adjustment for multiple comparisons using Benjamini-Hochberg correction. Two subacute PCVD subjects and three healthy controls did not have SOT data and were not included within linear regression analyses.

### 2.5. MR imaging acquisition

Subjects were scanned using a Siemens 3T Magnetom Prisma Fit equipped with a 32-channel head-only coil.

### 2.6. DWI protocol

Multi-shell diffusion weighted imaging (DWI) was obtained with optimal angular coverage using 128 diffusion directions distributed over 4 shells (4 volumes of b = 300 s/mm^2^, 17 of b = 650, 39 of b = 1,000, and 68 volumes of b = 2,000 s/mm^2^), with 2 mm^3^ isotropic voxel resolution, multiband factor 3, TE = 79 ms, TR = 2,750 ms, flip angle = 78°, AP phase encode (PE) direction = AP, and 232 × 256 FOV. Additionally, 12 b = 0 s/mm^2^ images were acquired interspersed between the diffusion volumes. We also acquired two volumes of b = 0 s/mm^2^ in the opposite PE direction to correct for distortion and other artifacts.

### 2.7. DWI preprocessing

Diffusion modeling preprocessing consisted of standard workflows in the FMRIB Software Library (FSL version 6.0: Wellcome Centre for Integrative Neuroimaging, Oxford, UK), including “topup” (Andersson et al., 2003; Smith et al., 2004) for susceptibility-induced distortion correction and “eddy” (Andersson et al., 2003, 2016, 2017; Andersson & Sotiropoulos, 2016) for eddy current and subject motion correction. Brain extraction was then performed using BET (Smith, 2002). Quality control of preprocessed data was assessed using Eddy QC (Bastiani et al., 2019) with manual inspection.

### 2.8. Fitting diffusion tensors and multicompartment modeling using NODDI

DTIFIT (https://fsl.fmrib.ox.ac.uk/fsl/fslwiki/FDT/UserGuide#DTIFIT) was run on the preprocessed eddy-corrected data to fit a tensor model to raw diffusion data, generating eigenvalues of the diffusion tensor matrix (λ_1_, λ_2_, λ_3_), fractional anisotropy (FA), and mean diffusivity (MD). Maps of radial (RD: λ_23 =_ (λ_2 +_ λ_3_)/2) and axonal diffusivity (AD: λ_1_) were subsequently calculated.

Multiple compartment modeling was performed using NODDI (Tariq et al., 2016; Zhang et al., 2012). To do so, a GPU-enabled CUDA diffusion modelling toolbox (cudiMOT) was used to generate Bingham-NODDI maps (Tariq et al., 2016), including V_In_, V_Iso_, DA_B_, ODI_T_, ODI_S_, and ODI_P_. Compared to the conventional Watson-NODDI model, which assumes isotropic dispersion of neurites (Zhang et al., 2012), Bingham-NODDI provides a better estimation of the anisotropic orientation dispersion commonly encountered within regions of fanning and bending (Tariq et al., 2016).

### 2.9. Tract-based spatial statistics (TBSS)

Voxelwise statistics were performed on conventional DTI and NODDI parameters using TBSS (Smith et al., 2006). FA maps from each subject were aligned into a common space (MNI152 standard space) using the nonlinear registration tool FNIRT (https://fsl.fmrib.ox.ac.uk/fsl/fslwiki/FNIRT). Mean FA images were created and thinned to create a mean FA skeleton representing the center of common tracts and then thresholded at 0.3 to filter out areas of low FA or regions with high variability across subjects. Each subject’s aligned FA data was then projected into the skeleton, and the resulting data were subjected to voxelwise cross-subject statistics. The FA-based nonlinear warps and skeleton projection were also applied to all other non-FA maps derived from DTI and NODDI fitting.

Injured and control subjects were compared with a group-level, voxelwise analysis performed on skeletonized diffusion metric maps using TBSS (Smith et al., 2006). Comparisons between subacute PCVD and controls were performed using voxelwise GLM analysis adjusted for age and corrected for multiple comparisons using familywise error rate (FWE) estimates at P_FWE_ ≤ .05 by permutation testing using the “randomise” tool in FSL (Winkler et al., 2014) with threshold-free cluster enhancement (TFCE) (Smith & Nichols, 2009).

### 2.10. Relationship between clinical measures and ROI-extracted diffusion metrics

First, binary mask images for regions of interest (ROIs) corresponding to white matter tracts were generated based on the Johns Hopkins University ICBM-DTI-81 WM labels atlas (Mori et al., 2008), which has been used in numerous prior concussion studies (Eierud et al., 2014). Using these ROI-based binary masks, mean values for both DTI and NODDI metrics were obtained from each subject’s white matter skeleton. Right- and left-hemispheric ROIs were included as separate areas (32 ROIs in total).

To assess the relationship between specific neuroanatomical white-matter substrates and clinical measures, for each clinical measure and each ROI, we fit a linear regression with the clinical measure as the response variable and the diffusion metric, injury status, that is, healthy control versus PCVD, and their interaction as predictors, while adjusting for age, time since injury, and gender. Time since injury was log-transformed. The maximum value for “days since injury” from the PCVD group was used for healthy controls. The associations between clinical measures and ROI-extracted diffusion measures specifically affected by injury status were assessed by looking at the interaction between diffusion measure and injury status.

Linear regression analyses were performed on all measured clinical data, including subjective clinical vestibular measures, objective clinical vestibular measures, and non-vestibular clinical measures. Prior to linear regression, clinical measure and diffusion metric values were standardized. Three separate groups of models were created, one for each category of clinical measures: (i) subjective clinical vestibular measures (DHI, VVAS, & VOMS [composite and individual submeasures]; 3,200 models total); (ii) objective clinical vestibular measures using SOT measures (SOT 2, SOT 3, & SOM; 960 models total); and (iii) non-vestibular clinical measures (PCCS, BAI, & BDI; 960 models total). Upon inspection of the residuals, the residuals from the control group tended to have a lower variance than the residuals from the patient group, resulting in heteroscedasticity. This can lead to inaccurate P values. To address this issue, linear regression models were refit using robust regression with heteroscedasticity consistent errors, using the “HC3” option for improved accuracy in smaller samples (using the *sandwhich* package in R) (Long & Ervin, 2000). After removing the intercept term, multiple comparisons were corrected with FDR P < .05 within each group of models. Variance inflation factors for the interaction term in all models were assessed and determined to be less than five.

### 2.11. Statistics

All statistical analysis was performed within either FSL or R-4.2.2 software (r-project.org, R Foundation for Statistical Computing, Vienna, Austria) (R Core Team, 2022).

## 3. RESULTS

### 3.1. Demographic and clinical data

The present study included 37 healthy control (HC) subjects without a history of PCVD (19 females; median age 28 years old [IQR = 5]) and 23 subacute PCVD patients (12 females; median age 22 years old [IQR = 5]) (Table 1). There was nearly equal representation of sex within both groups (51% females in HC, and 52% females in ST). The injured group (PCVD) had a statistically significant lower median age of 22 years (IQR 5) compared to controls’ median age of 28 years old (IQR 5) (P < .0001). The median length of time since concussion for the PCVD group was 35 days (IQR 23). Mechanism of injury predominantly included sports-related concussions (57%) and automobile accidents (30%) (Table 1).

Subjects underwent clinical testing using both subjective self-reported and objective clinical measures. Self-reported clinical measures included non-vestibular (PCSS, BDI, & BAI) and vestibular-based assessments (DHI, VVAS, & VOMS). Group-wise differences using Mann-Whitney rank sum tests revealed significantly greater self-reported symptoms (higher scores) across all clinical measures within the injured group (PCVD) compared to healthy controls (Table 1). Subjects also underwent objective quantitative dynamic posturography assessment using the Sensory Organization Test (SOT). Group-wise differences using Mann-Whitney rank sum tests revealed greater sway variability in conditions (lower) SOT scores in condition 2, that is, eyes-closed, fixed surface (effect size *r* = 0.40; P = .022) and condition 3, that is, sway-referenced visual surround (effect size *r* = 0.37; P = .022) within PCVD subjects compared to healthy controls. The PCVD group also had a lower somatosensory ratio relative to controls (effect size *r* = 0.38; P = .022). No differences were seen between treatment groups for the other SOT conditions (SOT 1, 4, 5, and 6, and composite SOT).

### 3.2. Group-wise TBSS

**Fig. 1.**
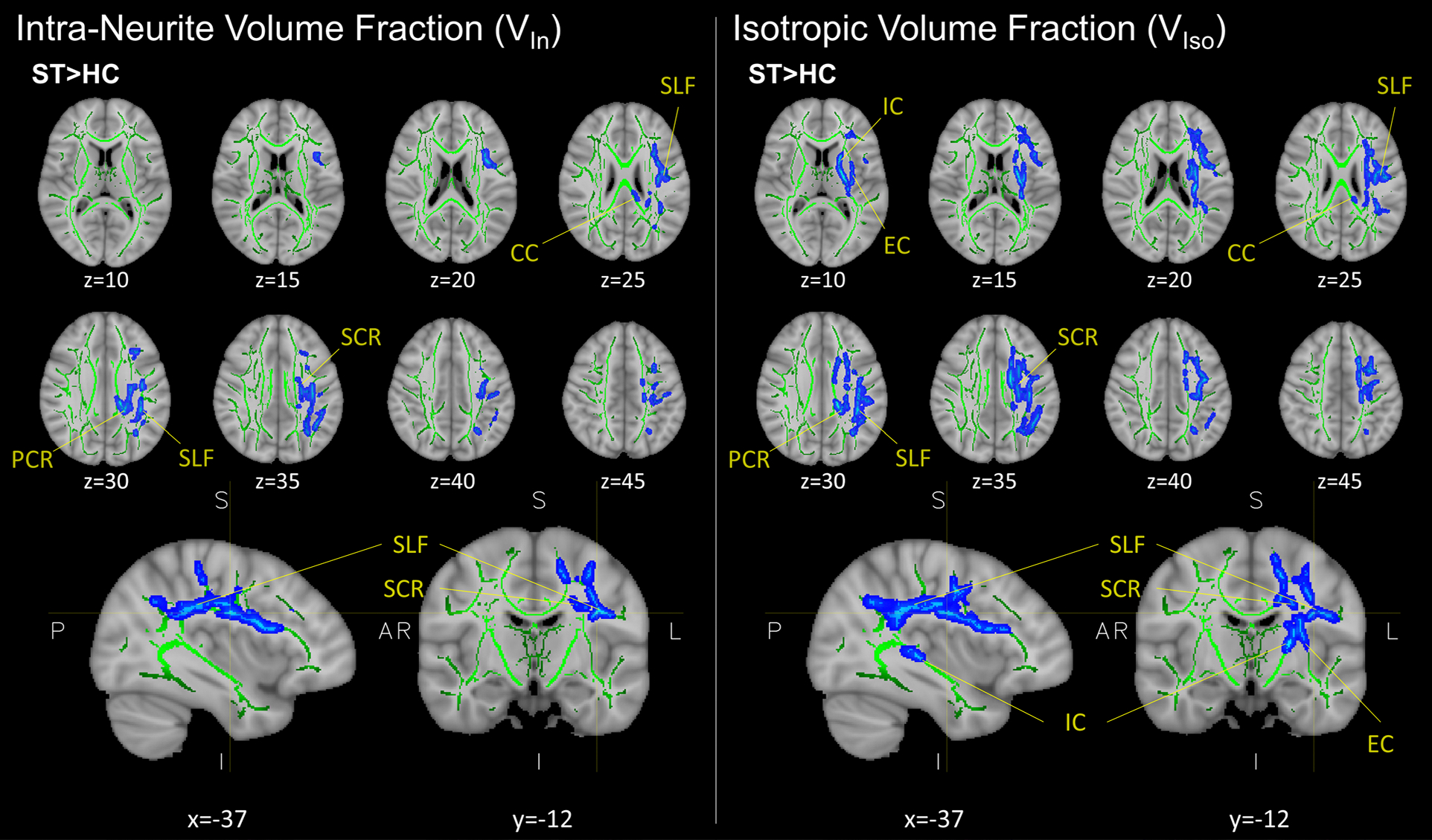
Representative results from group-level cluster-based (voxelwise) analysis of NODDI metrics V_In_ and V_Iso_ between control (HC; n = 37) and subacute postconcussive vestibular dysfunction (PCVD) patients (ST; n = 23) using tract-based spatial statistics. Regions in blue (seen as “fattened” results) correspond to areas of higher V_In_ and V_Iso_ in PCVD group relative to control. The mean FA skeleton (green) is overlaid on the FA158 T1 brain. Results corrected for multiple comparisons across space using TFCE-FWE (P < .05); representative images shown in radiographic orientation with MNI coordinates below. V_In_, intra-neurite volume fraction; V_Iso_, isotropic volume fraction; SCR, superior corona radiata; PCR, posterior corona radiata; SLF, superior longitudinal fasciculus; CC, corpus collosum; IC, internal capsule; EC, external capsule.

Voxelwise statistics revealed significant changes in two NODDI parameters: (1) the intracellular volume fraction (V_In_), also known as neurite density index (NDI), and (2) free water (isotropic) volume traction (V_Iso_). Voxelwise group-level analysis demonstrated increases in both V_In_ and V_Iso_ (P_FWE_ ≤ .05) within the subacute PCVD group compared to healthy controls (Fig. 1). Increases in V_In_ and V_Iso_ were found in overlapping anatomical regions within the left hemisphere, including the superior and posterior corona radiata, superior longitudinal fasciculus, and corpus callosum. Higher V_Iso_ was also seen within the internal and external capsules (Fig. 1). No other group-level differences were seen for the remaining DTI (FA, MD, AD, RD) or NODDI (DA_B_, ODI_T,P,S_) metrics (Table 2).

### 3.3. Relationship between ROI-extracted diffusion metrics and clinical vestibular measures

**Fig. 2.**
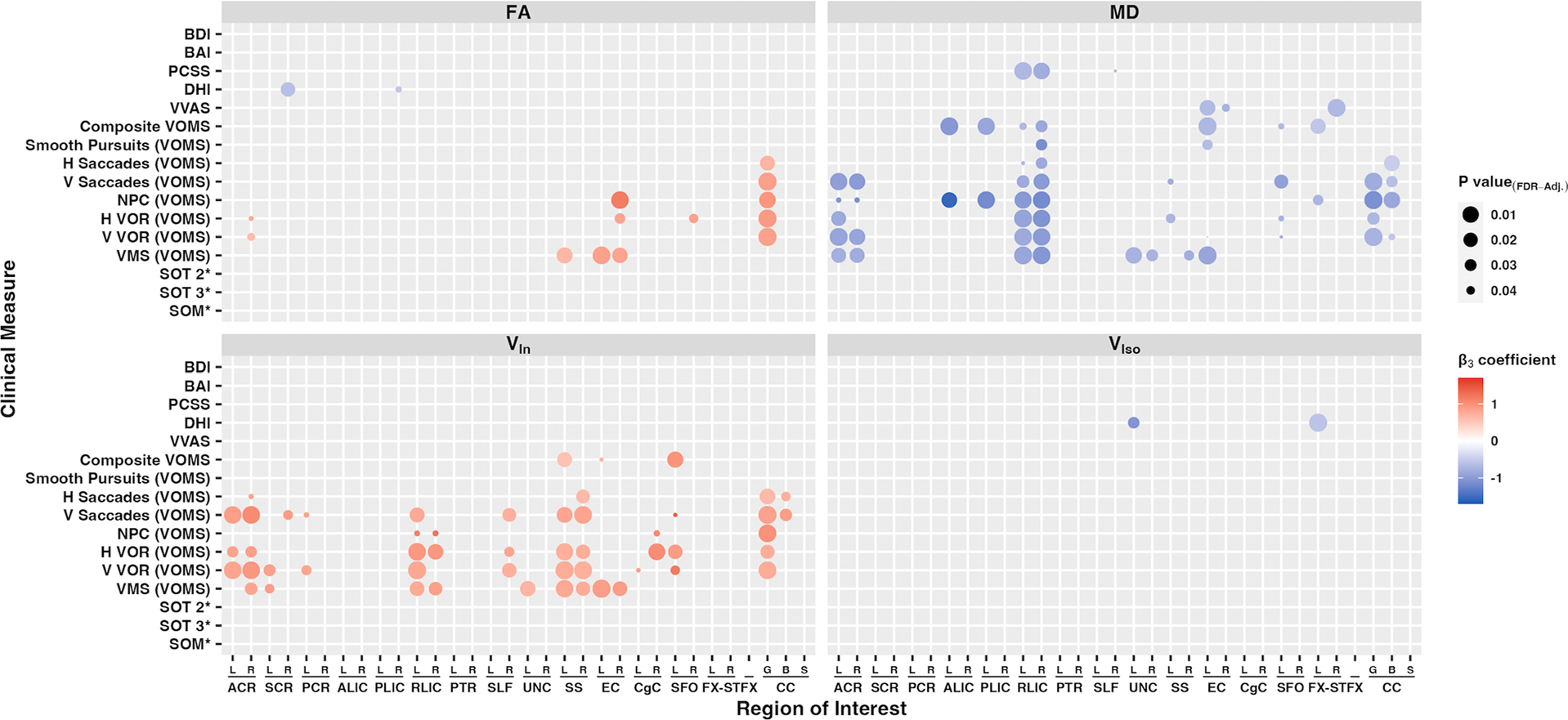
Heatmap plot of statistically significant standardized beta coefficients (**β_3_**) from multiple linear regression models comparing individual clinical measures (response variable) to ROI-extracted diffusion metrics (predictor variable) from the subacute PCVD group FA, MD, V_In_, and V_Iso_ diffusion metrics. The linear regression model is the following: Clinical Measure = β_0_ + β_1_* Diffusion Metric + β_2_* Injury Status + β_3_* (Diffusion Metric * Injury Status) + β_4_*log(days since concussion) + β_5_* Age + β_6_* Gender + error. **β_3_** corresponds to the beta coefficient for the interaction term between ROI-extracted diffusion metric and Injury status. Color denotes strength of relationship (red positive, blue negative), and size of circle corresponds to the inverse size of the FDR-adjusted P value. Three separate groups of multiple linear regression models were created using robust regression with heteroscedasticity consistent errors, one for each category of clinical measures: (i) subjective clinical vestibular measures (DHI, VVAS, VOMS [composite and individual submeasures]; 3,200 models total); (ii) objective clinical vestibular measures using SOT measures (SOT 2, SOT 3, and SOM; 960 models total); and (iii) non-vestibular clinical measures (PCCS, BAI, BDI; 960 models total). Other model variables include the log of “days since injury,” age, and gender. Multiple comparisons were adjusted with FDR P < .05. ROIs include areas from the JHU ICBM DTI-81 atlas. * Raw SOT scores (SOT 2, 3 and SOM) are positively corelated with better sensory organization (lower clinical severity). To match the pattern of other clinical measures, that is, higher score and higher clinical severity, the additive inverse is depicted for SOT measures (SOT 2*, SOT 3*, and SOM*). ACR, anterior corona radiata; SCR, superior corona radiata; PCR, posterior corona radiata; ALIC, anterior limb of internal capsule; PLIC, posterior limb of internal capsule; RLIC, retrolenticular part of the internal capsule; PTR, posterior thalamic radiation; SLF, superior longitudinal fasciculus; UNC, uncinate fasciculus; SS, sagittal stratum (inferior fronto-occipital fasciculus / inferior longitudinal fasciculus); EC, external capsule; CgC, cingulum (cingulate gyrus); SFO, superior fronto-occipital fasciculus; FX-ST, fornix-stria terminalis; CC, corpus callosum; laterality indicated with “R” or “L” for right and left, respectively. “G,” “B,” and “S” refer to genu, body, and splenium of the corpus callosum, respectively. BAI, Beck anxiety inventory; BDI, Beck depression inventory; PCVD, post-concussive vestibular dysfunction; PCSS, post-concussion symptom scale; DHI, dizziness handicap scale; VVAS, visual vertigo analog scale; SOT, sensory organization test; SOM, somatosensory; VIS, visual; VEST, vestibular; PREF, visual preference; VOMS, vestibular/ocular motor screening; NPC, near-point convergence; VOR, vestibular-ocular reflex; VMS, visual motion sensitivity; direction indicated with “H” or “V” for horizontal and vertical, respectively.

We used linear regression to assess the relationship between structural imaging findings and clinical vestibular measures, with an emphasis on the modification of the relationship between the DTI measure and clinical vestibular measure by disease status. Using the JHU DTI-81-ICBM atlas, 32 white-matter tract ROIs chosen based on prior studies (Muller et al., 2021) were used to extract mean values from each subject’s skeletonized conventional DTI and NODDI diffusion map, as summarized in Figure 2 and Supplementary Figure 1. A more detailed list is shown in Supplementary Tables 1-2. Injury status modifies the effect of ROI-extracted diffusion metrics (dMRI measure) on clinical vestibular measures. As expected, there is little relationship between dMRI measures and self-reported clinical measures in the healthy control group (main effect of dMRI measure P > .05). There is a negative interaction with injury status (PCVD) with MD and RD in numerous regions of interest (Fig. 2 & Supplementary Fig. 1), and positive interactions with FA and V_In_ in numerous regions. The ROIs associated with self-reported clinical measures with MD and RD also showed areas of significant overlap with V_In_. Specific ROIs that were associated with self-reported symptoms included areas seen in the TBSS results, such as the superior and posterior corona radiata, and corpus collusum.

Associations between diffusion metrics and objective clinical measures were performed using SOT measures that significantly differed between PCVD and healthy controls, including SOT 2, SOT 3, and SOM. No significant associations between diffusion measures and objective clinical measures were found upon correction for multiple comparisons (Fig. 2 & Supplementary Fig. 1). Despite changes in V_Iso_ being detected within the whole-brain analysis, only two associations existed between clinical measures and ROI-extracted V_Iso_ metrics (Fig. 2).

**Fig. 3.**
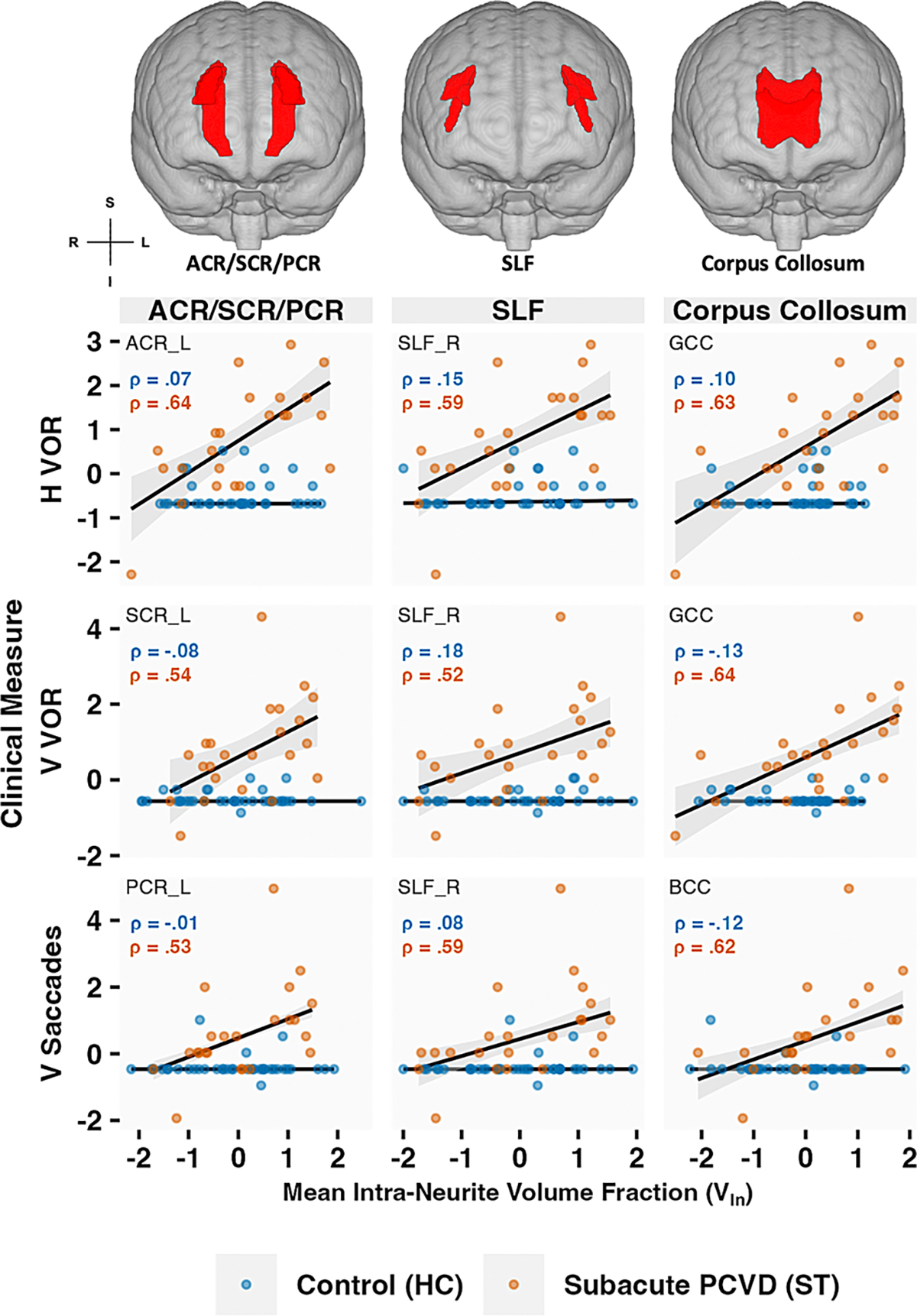
Representative scatter plots of standardized VOMS clinical submeasures and ROI-extracted and averaged NODDI metric, intra-neurite volume fraction (V_In_) for both healthy controls (HC) and subacute PCVD (ST). VOMS clinical measures include VOMS sub-measures (H & V VOR, and V Saccades). Spearman correlation coefficients are shown for descriptive purposes. Representative ROIs include the body and genu of the corpus callosum (BCC & GCC), corona radiata, and SLF, derived from the JHU ICBM DTI-81 atlas. For each plot, a straight-line linear model trendline with 95% confidence interval band is included. ACR, anterior corona radiata; SCR, superior corona radiata; PCR, posterior corona radiata; SLF, superior longitudinal fasciculus; laterally denoted by `_R` or `_L` for right and left respectively; `GCC` and `BCC` refer to genu and body of the corpus callosum, respectively.

To support our main analysis of the interaction effects, we conducted a post-hoc exploratory analysis with simple Spearman correlations between clinical measures and the DTI measures for the interaction terms that were significant, which revealed a positive relationship between V_In_ and VOMS within the corona radiata (ACR, SCR & PCR), SLF and CC (Fig. 3).

## 4. DISCUSSION

The present study utilized a complimentary approach with both conventional DTI and NODDI diffusion MRI to assess white matter microstructure within subacute PCVD and its relationship to clinical vestibular symptoms. Important findings within the present study revealed significant increases in two NODDI metrics, V_In_ (intraneurite volume fraction) and V_Iso_ (isotropic volume fraction), within overlapping key white matter tracts in the left hemisphere of patients with subacute PCVD, including the corpus collosum, SLF, SCR, and PCR. Additionally, linear regression analysis using ROI-extracted averaged diffusion measures revealed significant associations between several of these affected WM areas and self-reported clinical vestibular measures, including the SCR. Our findings implicating the SCR and SLF within subacute PCVD further support prior studies demonstrating their role in vestibular function and dysfunction (Gattu et al., 2016; Hadi et al., 2022; Kahane et al., 2003; Spena et al., 2006). The SCR and SLF represent large WM fiber bundles involved in multimodal processing (Hadi et al., 2022; Kamali et al., 2014; Mori et al., 2008; Spena et al., 2006). Thus, perturbation to these structures may thwart sensory integration critical to vestibular function, potentially contributing to PCVD symptoms.

Although no voxelwise group-level differences were seen using DTI metrics, there were numerous significant associations between ROI-extracted DTI and clinical vestibular measures. Despite differences in V_Iso_ using group-level voxelwise analysis, few correlations were seen when assessing the relationship between ROI-extracted V_Iso_ averages and clinical measures. This is particularly interesting given that regions from MD and RD maps exhibited strong correlations with self-reported clinical vestibular measures. Of all clinical vestibular measures, VOMS, which is a provocative self-reported measure, appeared to show greater correlation with diffusion metrics compared to VVAS, DHI, and SOT.

This is the first study to our knowledge that has employed the use of NODDI to study postconcussive vestibular dysfunction within the subacute phase of injury. Although comparing conventional DTI to NODDI was not an explicit purpose of the current study, our results revealed significant changes in whole-brain voxelwise analysis comparing PCVD to controls using NODDI metrics, while none were seen with conventional DTI. This is in concordance with current literature demonstrating NODDI’s improved specificity for detecting neuropathology compared to conventional DTI (Muller et al., 2021; Palacios et al., 2020). This advantage of NODDI is attributed to its ability to model biophysical compartments, which include (i) intraneuritic regions, that is, axons and dendritic processes; (ii) extra-neuritic regions, that is, glia, neuronal cell bodies, ependymal cells, and vascular structures; and (iii) isotropic (free water) regions, that is, cerebrospinal fluid (Kamiya et al., 2020; Tariq et al., 2016; Zhang et al., 2012). Since its inception in 2012, NODDI has been used in conjunction with DTI to characterize white matter changes following concussion (Churchill et al., 2017, 2019; Muller et al., 2021; Palacios et al., 2020; Wu et al., 2018).

The significance of greater V_In_ within the present study, which corresponds to increased neurite (axonal) density, may be explained by axonal swelling secondary to persistent inflammation and/or remodeling of neurites in response to injury (Kamiya et al., 2020). This finding parallels a prior study by Churchill et al. in which increased V_In_ was found within individuals with chronic concussion (Churchill et al., 2017). In a more recent longitudinal study examining structural changes in individuals participating in full contact American football, Maged et al. (2023) demonstrated greater V_In_ over a period of 4 years post-injury in the right SLF of football players at high position-based impact risk. In contrast to these findings of greater V_In_ following injury, other reports have shown decreased V_In_ following concussion during the acute-subacute (Palacios et al., 2020; Wu et al., 2018) and chronic phase of injury (Muller et al., 2021; Palacios et al., 2020). Although age at injury is unlikely to be the sole factor explaining differences in findings across these studies, it is worth noting that Churchill et al. and our study involved mostly young adults within their 20 s, while subjects from Wu et al. (2018) and Palacios et al. (2020) were mostly within their 30 s, and those from Muller et al. (2021) were in their late 40 s. Other differences across these respective studies include mechanism of injury, with some limited to a single injury mechanism, such as automobile-based mTBI (Wu et al., 2018), and other studies involving either sports-related concussion (Churchill et al., 2017) or mixed-mechanism mTBI (Muller et al., 2021; Palacios et al., 2020), the latter similar to the present study. Lastly, the chronicity of injury also varied across studies as well, with Palacios et al. (2020) and Maged et al. (2023) being the only two longitudinal mTBI NODDI study, whereas others were cross-sectional studies limited to the acute-subacute (Churchill et al., 2019; Wu et al., 2018) or chronic phase of injury (Churchill et al., 2017; Muller et al., 2021).

Our study also revealed increases in V_Iso_, which is physiologically low in white matter regions, as it corresponds to free water (isotropic) regions like CSF. Greater V_Iso_ within white matter regions following concussion may reflect increased interstitial water or vasogenic edema secondary to inflammation (Palacios et al., 2020). In a longitudinal study by Palacios et al. (2020), increased V_Iso_ was associated with individuals who exhibited postconcussive symptoms. Despite seeing prominent voxelwise differences in V_Iso_ within the present study, there were substantially fewer significant associations between ROI-extracted V_Iso_ metrics and clinical measures within PCVD patients relative to V_In_, suggesting that V_In_ may be more sensitive to vestibular dysfunction. In addition to V_In_ and V_Iso_, we also examined potential differences in ODI, which corresponds to axonal dispersion seen in WM tracts, which is typically seen in WM regions of fanning and/or bending. We saw no differences in whole-brain voxelwise analysis of ODI (including total [ODI_T_], primary [ODI_P_] and secondary [ODI_S_]) between individuals with and without PCVD, although subsequent analysis using ROI-extracted mean values of ODI from the SCR revealed significant positive correlations with the dizziness handicap score. Several prior mTBI studies using NODDI have reported increased ODI (Churchill et al., 2019; Oehr et al., 2021) following concussion, while others saw decreased ODI (Churchill et al., 2017; Muller et al., 2021). This may reflect differences in white matter remodeling/reorganization following injury. Additionally, differences in study design again may also account for this finding. Together, this study reveals microstructural white matter features related to PCVD and further supports the use of multi-shell diffusion-based models, like that of NODDI, for detecting subtle, yet clinically significant pathologies.

Although this is the first report using NODDI to study structural changes underlying vestibular impairment following mTBI, there are several prior studies using conventional DTI metrics to characterize white matter microstructure in individuals with vestibular impairment following concussion (Alhilali et al., 2014; Calzolari et al., 2020; Gard et al., 2022; Jang et al., 2021). In a retrospective study examining white matter changes related to vestibulopathy, Alhilali et al. measured FA and MD in mTBI patients with and without vestibular impairment, including a subset of individuals with ocular convergence insufficiency. Compared to concussed patients without vestibulopathy, those with vestibular impairment exhibited decreased FA within the cerebellum and fusiform gyrus, and increased mean diffusivity within the cerebellum (Alhilali et al., 2014). Within the same study, concussion patients with ocular convergence insufficiency featured decreased FA within the right anterior thalamic radiations and right geneictulate nucleus optic tracts (Alhilali et al., 2014). Although there are few similarities in findings that exist between the Alhilali et al. (2014) study and our own, there are several important differences in design that require consideration. Alhilali et al. (2014) included patients ranging from 1 day post-injury to 486 days post-injury, whereas our injury cohort included subacute patients ranging from 14–131 days post-injury. As shown in prior studies, there are differences in reported DTI results between the acute, subacute, and chronic phases of injury, likely representative of different underlying pathophysiological processes (Kim et al., 2021). Additionally, individuals with vestulopathy within the acute phase who eventually recover within 10–14 days may structurally appear different on imaging than individuals with persistant vestibulopathy within the subacute and chronic phase of injury. Furthermore, the comparison group in Alhilali et al. (2014) was a concussion group without vestibulopathy whereas our control group was healthy controls without prior concussion. Of note, in contrast to the current study, Alhilali et al. did not exlude possible causes of peripheral vestibular impairment within their study.

In a more recent study examining patients with chronic sports-related post-concussive vestibular dysfunction (>6 months post-injury), Gard et al. (2022) utilized DTI and DKI to examine cerebellar white matter microstructure. Similar to the current study, there were no statistically significant differences in conventional DTI metrics. However, significant changes in DKI metrics were found. Notably, decreases in mean kurtosis (MK) and radial kurtosis (RK) were seen in the superior cerebellar peduncle (Gard et al., 2022), which is thought to correspond to a loss of cellular structure (Gard et al., 2022). In another study within the subacute-chronic timeframe post-injury, Jang et al. (2021) also found no differences in DTI metrics between injured and healthy controls. However, using tractography to measure structural connectivity, Jang et al. (2021) demonstrated decreased tract volume of the core vestibular projection (CVP) in a subset of concussed individuals. The CVP is a white matter tract connecting the parieto-insular vestibular cortex (PIVC) and vestibular nuclei. Moreover, the decreased CVP tract volume was correlated with self-reported symptom severity as measured using the RPQ dizziness scores (Jang et al., 2021).

The use of vestibular-specific thalamo-cortical WM tracts within Jang et al. raises a weakness of the present study which utilized the more accessible, yet less anatomically and functionally specific JHU ICBM DTI-81 WM atlas. Further investigation into WM tracts associated with the core human vestibular cortex, principally the intraparietal sulcus (PIC) and parietal operculum 2 (OP2) (Indovina et al., 2020; Wirth et al., 2018), will help provide more detailed vestibular-specific characterization of anatomical substrates purturbed following injury. Furthermore, given the cross-sectional nature of the present study, it is difficult to determine whether these intra-neurite and free water WM changes seen within the PCVD group are indicative of direct trauma to these regions or a response to injury, or if these differences existed prior to injury and thus potentially contributed towards vulnerability to symptoms post-injury. Future longitudinal repeated-measures designed studies will help resolve this question. An additional consideration for future research that applies not only to the study of PCVD but to TBI research as a whole pertains to the use of healthy subjects as control subjects. As discussed by other groups, incorperating orthopedic injury controls (Wilde et al., 2019) as well as recovered concussed controls (Stenberg et al., 2021) and control subjects with persistent non-vestibular-related impairment following concussion should be a priority for future investigation.

This study reveals microstructural white matter features related to PCVD and further supports the use of multi-shell diffusion-based models, like that of NODDI, for detecting subtle, yet clinically significant pathologies not otherwise discernable using conventional DTI. Future study considerations should keep in mind the heterogenous nature of concussion, which is likely attributed to, but not limited to variability in mechanism of injury, time since injury, and age at injury as well as to the specific postconcussive symptoms (Kim et al., 2021; Shah & Allen, 2017). These injury and demographic factors, along with underlying genetic and environmental predisposition, also likely contribute to individual risk of persistant symptoms following concusion and may be associated with unique anatomical pathologies.

## 5. CONCLUSIONS

While research has increasingly focused on examining changes within white matter following concussion, there has been limited attention on individuals with persistent symptoms, in particular vestibular dysfunction. Furthermore, given the heterogeneity of head injury, generalizations of neuroimaging related to concussion pose several important challenges. Instead, there is a need to identify underlying pathological changes within the brain that correspond to particular symptoms underlying postconcussive syndromes like that of PCVD. The present study revealed significant differences in NODDI metrics that correlated with vestibular symptoms. Further utilization of multimodal neuroimaging will provide diagnostic and prognostic value that will likely help inform therapeutic interventions targeting specific pathologies.

## Supplementary Material

Suppl

## Figures and Tables

**Table 1.** Demographics and clinical measure summary.

	Healthy control	Subacute PCVD	n		P value*
	(HC)	(ST)	HC	ST	HC vs ST

Demographic					
N	37	23			-
Female	19 (51%)	12 (52%)			-
Age, years	28.0 (5.0) [22–50]	22.0 (5.0) [16–34]			P < .0001
Days since concussion	-	35.0 (23.0) [14–131]			-
Mechanism of Injury					
Sports-Related	-	13 (57%)			-
Automobile-Related	-	7 (30%)			-
Other	-	3 (13%)			-
Non-Vestibular Clinical Measures					
PCSS	0.0 (0.0)	32.0 (32.0)	27	21	P < .00001
BAI	2.0 (5.5)	10.0 (10.0)	35	18	P < .0001
BDI	3.0 (7.0)	12.0 (10.2)	35	18	P < .0001
Subjective Vestibular Clinical Measures					
DHI	0.0 (0.0)	36.0 (18.0)	35	19	P < .00001
VVAS	0.0 (1.8)	20.3 (27.5)	35	19	P < .00001
VOMS (minus baseline)					
Composite VOMS	0.0 (2.0)	15.0 (18.0)	37	20	P < .00001
Smooth Pursuits	0.0 (0.0)	0.0 (1.0)	37	23	P < .01
Horizontal Saccades	0.0 (0.0)	1.0 (1.8)	37	22	P < .00001
Vertical Saccades	0.0 (0.0)	2.0 (2.0)	37	23	P < .00001
NPC	0.0 (0.0)	2.0 (3.0)	37	21	P < .0001
NPC Avg (cm)	2.3 (2.7)	5.3 (6.3)	37	23	P < .01
Horizontal VOR	0.0 (1.0)	4.0 (3.5)	37	23	P < .00001
Vertical VOR	0.0 (0.0)	4.0 (4.5)	37	23	P < .00001
VMS	0.0 (1.0)	4.0 (5.0)	37	23	P < .00001
SOT					
Composite SOT	78.5 (7.5)	75.0 (11.0)	34	21	n.s.
SOT Condition #1	92.5 (2.7)	92.0 (6.0)	34	21	n.s.
SOT Condition #2	92.7 (2.8)	90.7 (11.3)	34	21	P = .022
SOT Condition #3	93.3 (2.0)	89.3 (8.3)	34	21	P = .022
SOT Condition #4	75.5 (9.8)	75.7 (12.0)	34	21	n.s.
SOT Condition #5	67.5 (10.9)	62.8 (24.5)	34	20	n.s.
SOT Condition #6	71.8 (8.8)	66.3 (17.7)	34	20	n.s.
Sensory Analysis Ratios					
SOM	1.00 (0.03)	0.97 (0.07)	34	21	P = .022
VIS	0.83 (0.09)	0.83 (0.12)	34	21	n.s.
VEST	0.73 (0.11)	0.68 (0.23)	34	20	n.s.
PREF	1.02 (0.06)	1.01 (0.06)	34	20	n.s.

Note, unless otherwise indicated, values correspond to median with interquartile range (IQR) within parenthesis; range indicated within brackets.

* Mann-Whitney U test w/ BH correction.

BAI, Beck anxiety inventory; BDI, Beck depression inventory; PCVD, post-concussive vestibular dysfunction; PCSS, post-concussion symptom scale; DHI, dizziness handicap scale; VVAS, visual vertigo analog scale; SOT, sensory organization test; SOM, somatosensory; VIS, visual; VEST, vestibular; PREF, visual preference; VOMS, vestibular/ocular motor screening; NPC, near-point convergence; VOR, vestibular-ocular reflex; VMS, visual motion sensitivity.

**Table 2.** Summary of between-group voxelwise analysis using TBSS.

	P value*	
Diffusion metric	HC > ST	ST > HC

DTI		
FA	P = .898	P = .108
MD	P = .067	P = .992
AD	P = .306	P = .899
RD	P = .067	P = .990
NODDI		
V_In_	P = .672	**P = .046**
V_Iso_	P = .902	**P = .021**
ODI_P_	P = .886	P = .584
ODI_S_	P = .262	P = .902
ODI_T_	P = .388	P = .574
DA	P = .961	P = .257

Nonparametric unpaired t-tests were used to compare group differences between healthy controls (HC) and subacute PCVD patients (ST), with age included as a nuisance variable. Bolded values correspond to P values < .05.

* Familywise error (FWE) corrected for multiple comparisons across space using Threshold-Free Cluster Enhancement.

TBSS, tract-based spatial statistics (TBSS); FA, fractional anisotropy; MD, mean diffusivity; RD, radial diffusivity; AD, axial diffusivity; V_In_, intraneurite volume fraction; V_Iso_, isotropic volume fraction; ODI_T/P/S_, orientation dispersion index_Total/Primary/Secondary_; DA, dispersion anisotropy.

## Data Availability

De-identified derived data supporting the findings of this study are available from the corresponding author (JWA) upon reasonable request. Data requests should include a formal project outline and may be subject to approval from the requestor’s local ethics committee and a formal data sharing and attribution agreement.
