# Supplementary material for: Assessment of white matter microstructure integrity in subacute postconcussive vestibular dysfunction using NODDI
Source: Imaging Neurosci (Camb). Author manuscript; Available in PMC 2025 Jul 31. (PMC12247550; doi:10.1162/imag_a_00147)
Supplement: Suppl [file NIHMS2035015-supplement-Suppl.pdf]

Supplementary Materials for *IMAG-23-0072*

Assessment of white matter microstructure integrity in subacute postconcussive vestibular dysfunction using NODDI

Joseph A. Behnke, MD, PhD[1], Vishwadeep Ahluwalia, PhD[2,3], Jeremy L. Smith, PhD, MSDS[1], Benjamin B. Risk, PhD[4], Jianna Lin, BS[5], Russell K. Gore, MD[6,7], Jason W. Allen, MD, PhD[8,*]

[1]Department of Radiology and Imaging Sciences, Emory University School of Medicine, Atlanta, Georgia, USA
[2]Georgia Institute of Technology, Atlanta, Georgia, USA
[3]GSU/GT Center for Advanced Brain Imaging, Atlanta, Georgia, USA
[4]Department of Biostatistics and Bioinformatics, Rollins School of Public Health, Emory University, Atlanta, GA, USA
[5]University of Central Florida College of Medicine, Orlando, Florida, USA
[6]Shepherd Center, Atlanta, Georgia, USA
[7]Wallace H. Coulter Department of Biomedical Engineering, Georgia Institute of Technology and Emory University, Atlanta, Georgia, USA
[8]Department of Radiology & Imaging Sciences, Indiana University School of Medicine, Indianapolis, Indiana, USA
*To whom correspondence should be addressed; email: allenjaw@iu.edu. Postal address: Department of Radiology and Imaging Sciences, Indiana University School of Medicine, IU Health University Hospital, 550 N. University Boulevard Indianapolis, IN, USA

I. **Supplementary Figure 1**
II. **Supplementary Table 1**
III. **Supplementary Table 2**

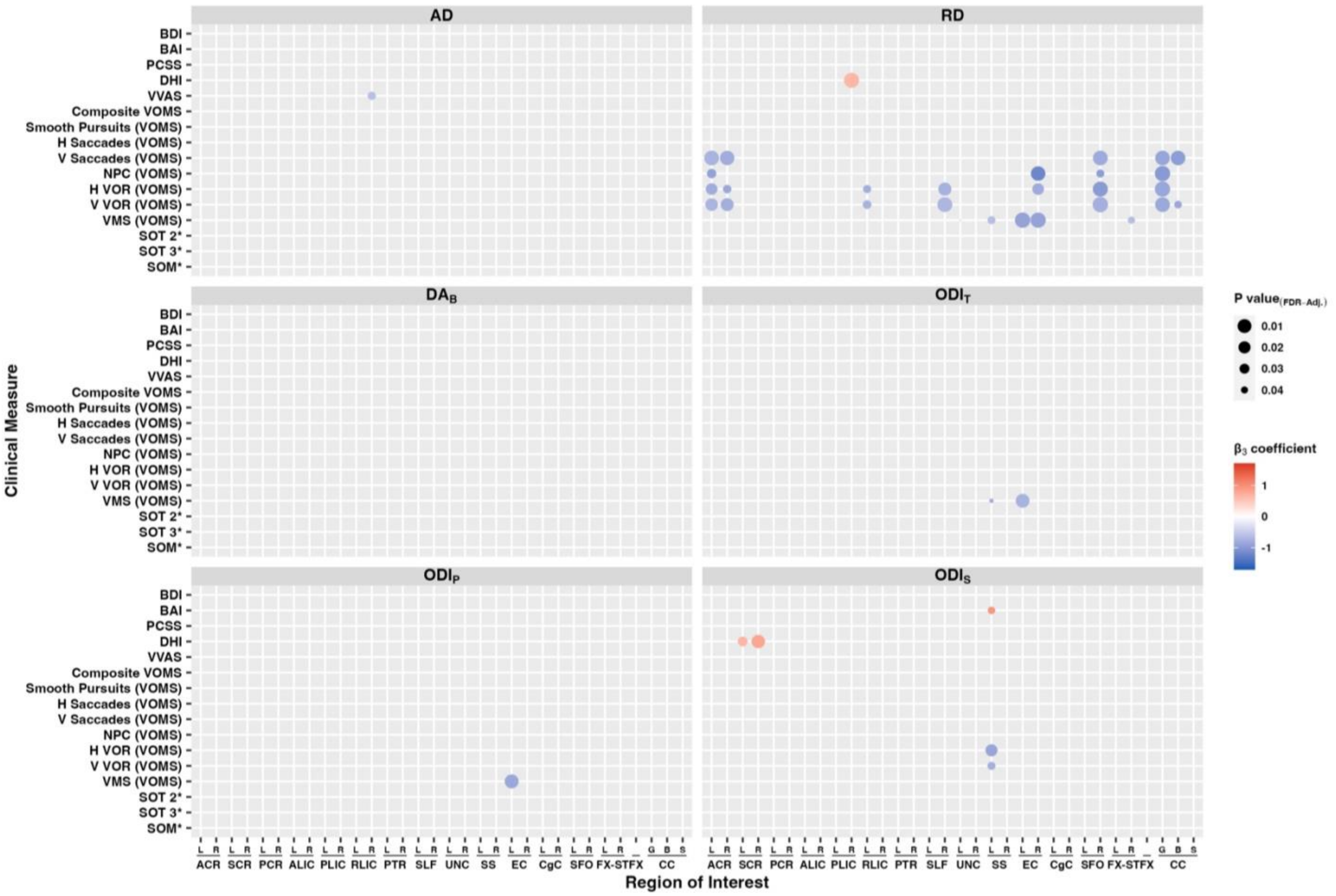

Supplementary Figure 1 *Heatmap plot of statistically significant standardized beta coefficients (*$\boldsymbol{\beta_3}$*) from multiple linear regression models comparing individual clinical measures (response variable) to ROI-extracted diffusion metrics (predictor variable) from the subacute PCVD group from AD, RD, *$ODI_{T/P/S}$* and *DA_B* diffusion metrics. The linear regression model is the following: Clinical Measure = *β_0* + *β_1***Diffusion Metric + *β_2** Injury Status + *$\boldsymbol{\beta_3}$** (Diffusion Metric * Injury Status) + *β_4***log(days since concussion) + *β_5***Age + *β_6***Gender + error. *$\boldsymbol{\beta_3}$* corresponds to the beta coefficient for the interaction term between ROI-extracted Diffusion Metric and Injury Status. Color denotes strength of relationship (red positive, blue negative) and size of circle corresponds to the inverse size of the FDR-adjusted P value. Three separate multiple linear regression models were created using robust regression with heteroscedasticity consistent errors, one for each category of clinical measures: i) subjective clinical vestibular measures (DHI,VVAS, VOMS (composite and individual submeasures); 3,200 models total), ii) objective clinical vestibular measures using SOT measures (SOT 2, SOT 3 and SOM; 960 models total) iii) non-vestibular clinical measures (PCCS, BAI, BDI; 960 models total). Other model variables include the log of "days since injury", age and gender. Multiple comparisons were adjusted with FDR P < .05. ROIs include areas from the JHU ICBM DTI-81 atlas. * Raw SOT scores (SOT 2, 3 and SOM) are positively corelated with better sensory organization (lower clinical severity). To match the pattern of other clinical measures, i.e., higher score à higher clinical severity, the additive inverse is depicted for SOT measures (SOT 2*, SOT 3*, and SOM*). Abbreviations: ACR, anterior corona radiata; SCR, superior corona radiata; PCR, posterior corona radiata; ALIC, anterior limb of internal capsule; PLIC, posterior limb of internal capsule; RLIC, retrolenticular part of the internal capsule; PTR, posterior thalamic radiation; SLF, superior longitudinal fasciculus; UNC, uncinate fasciculus; SS , sagittal stratum (inferior fronto-occipital fasciculus / inferior longitudinal fasciculus); EC, external capsule; CgC, cingulum (cingulate gyrus); SFO, superior fronto-occipital fasciculus; FX-ST, fornix-stria terminalis; CC, corpus callosum; laterality indicated with "R" or "L" for right and left, respectively. "G", "B" and "S" refer to genu, body and splenium of the corpus callosum, respectively. BAI, Beck anxiety inventory; BDI, Beck depression inventory; PCVD, post-concussive vestibular dysfunction; PCSS, post-concussion symptom scale; DHI, dizziness handicap scale; VVAS, visual vertigo analog scale; SOT, sensory organization test; SOM, somatosensory; VIS, visual; VEST, vestibular; PREF, visual preference; VOMS, vestibular/ocular motor screening; NPC, near-point convergence; VOR, vestibular-ocular reflex; VMS, visual motion sensitivity; direction indicated with "H" or "V" for horizontal and vertical, respectively*

Supplemental Table 1 Results of Robust Regression between Subjective Vestibular Clinical Measures (DHI, VVAS & VOMS) and Diffusion Measures

	Clinical Measure	Diffusion Metric	ROI	Robust Regression					Spearman Correlation Coefficient (ρ)	
				β	std. error	Adj. R^2	P $value_{unadj.}$	q.value	Ctrl	PCVD
1	DHI	FA	PLIC_R	-0.53	0.16	0.2	1.9e-03	4.5e-02	0.21	-0.26
2	DHI	FA	SCR_R	-0.62	0.17	0.27	6.4e-04	2.0e-02	0.25	-0.33
3	DHI	RD	PLIC_R	0.63	0.14	0.24	3.7e-05	2.3e-03	-0.2	0.32
4	DHI	V_{Iso}	FX-ST_L	-0.58	0.12	0.34	1.5e-05	1.2e-03	0.12	-0.62
5	DHI	V_{Iso}	UNC_L	-1.05	0.3	0.31	1.2e-03	3.1e-02	0.21	-0.45
6	DHI	ODI_S	SCR_L	0.65	0.19	0.24	1.2e-03	3.1e-02	-0.03	0.44
7	DHI	ODI_S	SCR_R	0.79	0.21	0.38	3.6e-04	1.3e-02	-0.09	0.65
8	VVAS	MD	EC_L	-0.66	0.17	0.19	4.0e-04	1.4e-02	0.04	-0.48
9	VVAS	MD	EC_R	-0.75	0.23	0.23	1.8e-03	4.1e-02	0.07	-0.45
10	VVAS	MD	FX-ST_R	-0.69	0.15	0.17	4.2e-05	2.5e-03	-0.24	-0.49
11	VVAS	AD	RLIC_R	-0.61	0.18	0.19	1.5e-03	3.6e-02	-0.07	-0.63
12	COMS Composite	MD	ALIC_L	-1.01	0.22	0.31	3.8e-05	2.3e-03	0.04	-0.47
13	COMS Composite	MD	EC_L	-0.7	0.14	0.25	1.1e-05	9.7e-04	0	-0.49
14	COMS Composite	MD	FX-ST_L	-0.56	0.15	0.19	4.7e-04	1.6e-02	-0.17	-0.63
15	COMS Composite	MD	PLIC_L	-0.88	0.21	0.36	1.3e-04	5.9e-03	0.02	-0.63
16	COMS Composite	MD	RLIC_L	-0.67	0.2	0.27	1.8e-03	4.3e-02	-0.11	-0.61
17	COMS Composite	MD	RLIC_R	-0.85	0.25	0.32	1.1e-03	3.0e-02	-0.12	-0.53
18	COMS Composite	MD	SFO_L	-0.69	0.21	0.23	2.0e-03	4.5e-02	0.2	-0.6
19	COMS Composite	V_{In}	EC_L	0.64	0.2	0.21	2.1e-03	4.7e-02	0.04	0.44
20	COMS Composite	V_{In}	SFO_L	0.97	0.25	0.33	3.2e-04	1.2e-02	-0.18	0.72
21	COMS Composite	V_{In}	SS_L	0.55	0.15	0.16	5.3e-04	1.7e-02	0.24	0.44
22	Smooth Pursuits	MD	EC_L	-0.65	0.19	0.09	1.4e-03	3.5e-02	0.08	-0.6
23	Smooth Pursuits	MD	RLIC_R	-1.12	0.33	0.27	1.2e-03	3.2e-02	-0.04	-0.71
24	H Saccades	FA	GCC	0.67	0.18	0.13	5.5e-04	1.8e-02	0.11	0.69
25	H Saccades	MD	BCC	-0.48	0.13	0.06	3.6e-04	1.3e-02	-0.09	-0.57
26	H Saccades	MD	RLIC_L	-0.67	0.21	0.12	2.1e-03	4.8e-02	-0.03	-0.75
27	H Saccades	MD	RLIC_R	-0.84	0.24	0.16	1.2e-03	3.1e-02	0.04	-0.74
28	H Saccades	V_{In}	ACR_R	0.84	0.26	0.19	2.1e-03	4.6e-02	-0.19	0.68
29	H Saccades	V_{In}	BCC	0.72	0.22	0.13	1.6e-03	3.8e-02	-0.03	0.7
30	H Saccades	V_{In}	GCC	0.63	0.16	0.12	3.9e-04	1.4e-02	-0.06	0.77
31	H Saccades	V_{In}	SS_R	0.63	0.17	0.11	7.5e-04	2.2e-02	-0.27	0.61
32	V Saccades	FA	GCC	0.86	0.17	0.24	5.4e-06	6.0e-04	0.08	0.66
33	V Saccades	MD	ACR_L	-0.95	0.23	0.29	1.2e-04	5.5e-03	0.16	-0.65
34	V Saccades	MD	ACR_R	-0.99	0.26	0.32	2.9e-04	1.1e-02	0.04	-0.66
35	V Saccades	MD	BCC	-0.62	0.18	0.12	1.2e-03	3.2e-02	-0.01	-0.48
36	V Saccades	MD	GCC	-0.82	0.18	0.22	4.2e-05	2.4e-03	0.06	-0.62
37	V Saccades	MD	RLIC_L	-0.83	0.24	0.22	9.8e-04	2.7e-02	-0.13	-0.78
38	V Saccades	MD	RLIC_R	-0.99	0.26	0.25	3.5e-04	1.3e-02	-0.02	-0.69
39	V Saccades	MD	SFO_L	-0.93	0.26	0.24	7.2e-04	2.2e-02	0.2	-0.73
40	V Saccades	MD	SS_L	-0.81	0.25	0.19	2.0e-03	4.5e-02	-0.06	-0.67
41	V Saccades	RD	ACR_L	-0.72	0.17	0.17	9.1e-05	4.4e-03	0.05	-0.65
42	V Saccades	RD	ACR_R	-0.8	0.2	0.21	1.5e-04	6.5e-03	0.05	-0.67
43	V Saccades	RD	BCC	-0.95	0.23	0.27	1.3e-04	5.8e-03	0.05	-0.65
44	V Saccades	RD	GCC	-0.87	0.21	0.26	1.1e-04	5.3e-03	-0.03	-0.71
45	V Saccades	RD	SFO_R	-0.82	0.19	0.2	9.4e-05	4.5e-03	0.13	-0.69
46	V Saccades	V_{In}	ACR_L	0.87	0.21	0.23	9.0e-05	4.4e-03	-0.15	0.63
47	V Saccades	V_{In}	ACR_R	1.03	0.24	0.31	6.6e-05	3.5e-03	-0.13	0.64
48	V Saccades	V_{In}	BCC	0.88	0.25	0.21	1.0e-03	2.8e-02	-0.12	0.62
49	V Saccades	V_{In}	GCC	0.84	0.18	0.24	1.7e-05	1.2e-03	-0.12	0.7
50	V Saccades	V_{In}	PCR_L	0.81	0.25	0.15	2.0e-03	4.6e-02	-0.01	0.53
51	V Saccades	V_{In}	RLIC_L	0.77	0.21	0.18	5.3e-04	1.7e-02	0.03	0.74
52	V Saccades	V_{In}	SCR_R	0.89	0.27	0.18	1.5e-03	3.7e-02	-0.06	0.59
53	V Saccades	V_{In}	SFO_L	1.36	0.42	0.38	2.1e-03	4.7e-02	-0.28	0.75
54	V Saccades	V_{In}	SLF_R	0.72	0.2	0.14	7.7e-04	2.3e-02	0.08	0.59
55	V Saccades	V_{In}	SS_L	0.82	0.21	0.21	3.7e-04	1.3e-02	-0.03	0.7
56	V Saccades	V_{In}	SS_R	0.81	0.19	0.21	6.0e-05	3.2e-03	-0.09	0.68
57	NPC	FA	EC_R	1.16	0.27	0.48	9.2e-05	4.5e-03	-0.29	0.71
58	NPC	FA	GCC	0.95	0.24	0.29	2.4e-04	9.5e-03	-0.13	0.64
59	NPC	MD	ACR_L	-1.05	0.32	0.34	2.1e-03	4.6e-02	0.21	-0.67
60	NPC	MD	ACR_R	-1.08	0.33	0.35	2.1e-03	4.6e-02	0.15	-0.66
61	NPC	MD	ALIC_L	-1.55	0.41	0.39	4.3e-04	1.5e-02	0.29	-0.64
62	NPC	MD	BCC	-0.87	0.22	0.28	2.6e-04	9.9e-03	-0.18	-0.63

Abbreviations: GCC, Genu of corpus callosum; BCC, Body of corpus callosum; SCC, Splenium of corpus callosum; ALIC, Anterior limb of internal capsule; PLIC, Posterior limb of internal capsule; RLIC, ; Retrolenticular internal capsule; ACR, Anterior corona radiata; SCR, Superior corona radiata; PCR, Posterior corona radiata; PTR, Posterior thalamic radiation; SS (IFO/ILF), Sagittal stratum (Inferior fronto-occipital fasciculus / inferior longitudinal fasciculus); EC, External capsule; CgC, Cingulum (cingulate gyrus); SLF, Superior longitudinal fasciculus; SFO, Superior fronto-occipital fasciculus; UNC, Uncinate fasciculus; FX, Fornix; ST-FX, Stria Terminalis-Fornix; _R, right-sided; _L, left-sided. PCVD, post-concussive vestibular dysfunction; DHI, dizziness handicap scale; VVAS, visual vertigo analog scale; VOMS, vestibular/ocular motor screening; NPC, near-point convergence; VOR, vestibular-ocular reflex; VMS, visual motion sensitivity; H, horizontal; V, vertical. Multiple comparisons of robust regressions adjusted for using the false-discovery rate (FDR). FDR-adjusted P values are shown in the `q.value` column. Spearman correlation coefficients between standardized diffusion and clinical measures for the control and PCVD groups are included for descriptive purposes.

Supplemental Table 1 Results of Robust Regression between Subjective Vestibular Clinical Measures (DHI, VVAS & VOMS) and Diffusion Measures

	Clinical Measure	Diffusion Metric	ROI	Robust Regression					Spearman Correlation Coefficient (ρ)	
				β	std. error	Adj. R^2	P value$_{unadj.}$	q.value	Ctrl	PCVD
63	NPC	MD	FX-ST_L	-0.73	0.22	0.17	1.4e-03	3.5e-02	0.06	-0.59
64	NPC	MD	GCC	-1.1	0.21	0.41	4.0e-06	5.5e-04	0.05	-0.75
65	NPC	MD	PLIC_L	-1.15	0.27	0.32	9.4e-05	4.5e-03	0.24	-0.73
66	NPC	MD	RLIC_L	-1	0.24	0.33	1.4e-04	6.3e-03	-0.03	-0.81
67	NPC	MD	RLIC_R	-1.16	0.27	0.31	1.1e-04	4.9e-03	-0.04	-0.73
68	NPC	RD	ACR_L	-0.9	0.26	0.27	1.2e-03	3.2e-02	0.17	-0.66
69	NPC	RD	EC_R	-1.2	0.29	0.5	1.5e-04	6.5e-03	0.31	-0.73
70	NPC	RD	GCC	-0.99	0.2	0.33	1.1e-05	9.7e-04	0.13	-0.72
71	NPC	RD	SFO_R	-0.94	0.28	0.27	1.5e-03	3.7e-02	0.02	-0.69
72	NPC	V_{In}	CgC_R	1.04	0.32	0.24	1.9e-03	4.5e-02	-0.25	0.5
73	NPC	V_{In}	GCC	0.98	0.22	0.34	5.2e-05	2.9e-03	-0.17	0.72
74	NPC	V_{In}	RLIC_L	1.12	0.34	0.43	2.0e-03	4.5e-02	0.05	0.83
75	NPC	V_{In}	RLIC_R	1.23	0.38	0.39	2.0e-03	4.5e-02	0	0.71
76	H VOR	FA	ACR_R	0.78	0.24	0.26	2.1e-03	4.7e-02	0.09	0.61
77	H VOR	FA	EC_R	0.81	0.24	0.3	1.4e-03	3.4e-02	-0.19	0.59
78	H VOR	FA	GCC	0.9	0.19	0.38	1.5e-05	1.2e-03	0.05	0.65
79	H VOR	FA	SFO_R	0.84	0.25	0.29	1.6e-03	3.8e-02	-0.13	0.55
80	H VOR	MD	ACR_L	-0.84	0.23	0.29	5.5e-04	1.8e-02	-0.07	-0.61
81	H VOR	MD	GCC	-0.71	0.2	0.21	1.0e-03	2.8e-02	-0.15	-0.52
82	H VOR	MD	RLIC_L	-0.9	0.2	0.37	3.3e-05	2.1e-03	-0.18	-0.82
83	H VOR	MD	RLIC_R	-1.06	0.25	0.39	9.8e-05	4.7e-03	-0.1	-0.7
84	H VOR	MD	SFO_L	-0.75	0.23	0.19	2.0e-03	4.5e-02	0.06	-0.71
85	H VOR	MD	SS_L	-0.71	0.21	0.19	1.6e-03	3.8e-02	-0.24	-0.65
86	H VOR	RD	ACR_L	-0.8	0.22	0.31	7.4e-04	2.2e-02	-0.1	-0.7
87	H VOR	RD	ACR_R	-0.84	0.25	0.33	1.4e-03	3.6e-02	-0.06	-0.69
88	H VOR	RD	EC_R	-0.81	0.22	0.3	7.4e-04	2.2e-02	0.07	-0.6
89	H VOR	RD	GCC	-0.86	0.18	0.35	1.1e-05	9.4e-04	-0.06	-0.66
90	H VOR	RD	RLIC_L	-0.78	0.23	0.22	1.5e-03	3.7e-02	-0.05	-0.61
91	H VOR	RD	SFO_R	-0.98	0.22	0.4	4.2e-05	2.5e-03	0.09	-0.7
92	H VOR	RD	SLF_R	-0.75	0.2	0.23	4.2e-04	1.4e-02	-0.12	-0.6
93	H VOR	V_{In}	ACR_L	0.83	0.24	0.28	1.2e-03	3.2e-02	0.07	0.64
94	H VOR	V_{In}	ACR_R	0.87	0.25	0.28	1.2e-03	3.2e-02	0.14	0.59
95	H VOR	V_{In}	CgC_R	1.03	0.26	0.35	2.0e-04	8.2e-03	0.12	0.47
96	H VOR	V_{In}	GCC	0.74	0.21	0.26	7.0e-04	2.1e-02	0.1	0.63
97	H VOR	V_{In}	RLIC_L	0.92	0.22	0.39	8.1e-05	4.0e-03	0.06	0.77
98	H VOR	V_{In}	RLIC_R	0.94	0.25	0.32	4.3e-04	1.5e-02	0.13	0.62
99	H VOR	V_{In}	SFO_L	0.88	0.24	0.19	6.1e-04	1.9e-02	-0.08	0.68
100	H VOR	V_{In}	SLF_R	0.82	0.24	0.25	1.4e-03	3.6e-02	0.15	0.59
101	H VOR	V_{In}	SS_L	0.72	0.18	0.22	1.3e-04	6.0e-03	0.25	0.69
102	H VOR	V_{In}	SS_R	0.73	0.2	0.23	6.4e-04	2.0e-02	0.2	0.67
103	H VOR	ODI_S	SS_L	-0.87	0.24	0.24	6.5e-04	2.0e-02	-0.01	-0.59
104	V VOR	FA	ACR_R	0.61	0.19	0.14	1.8e-03	4.2e-02	0.01	0.56
105	V VOR	FA	GCC	0.84	0.17	0.29	5.5e-06	6.1e-04	-0.08	0.64
106	V VOR	MD	ACR_L	-0.89	0.2	0.31	3.1e-05	2.0e-03	0.23	-0.58
107	V VOR	MD	ACR_R	-0.89	0.23	0.3	3.0e-04	1.1e-02	0.17	-0.57
108	V VOR	MD	BCC	-0.55	0.17	0.11	1.9e-03	4.4e-02	0.07	-0.4
109	V VOR	MD	EC_L	-0.6	0.19	0.11	2.2e-03	4.9e-02	0.13	-0.53
110	V VOR	MD	GCC	-0.74	0.15	0.21	4.9e-06	5.6e-04	0.12	-0.56
111	V VOR	MD	RLIC_L	-0.84	0.19	0.28	7.3e-05	3.7e-03	0.03	-0.69
112	V VOR	MD	RLIC_R	-0.98	0.23	0.29	9.6e-05	4.6e-03	0.03	-0.63
113	V VOR	MD	SFO_L	-0.8	0.25	0.21	2.2e-03	4.8e-02	0.31	-0.62
114	V VOR	RD	ACR_L	-0.72	0.2	0.21	5.3e-04	1.7e-02	0.08	-0.65
115	V VOR	RD	ACR_R	-0.78	0.21	0.24	4.6e-04	1.5e-02	0.06	-0.64
116	V VOR	RD	BCC	-0.83	0.25	0.25	1.6e-03	3.8e-02	0.12	-0.57
117	V VOR	RD	GCC	-0.84	0.2	0.29	8.3e-05	4.1e-03	0.1	-0.68
118	V VOR	RD	RLIC_L	-0.74	0.22	0.18	1.4e-03	3.5e-02	0.09	-0.56
119	V VOR	RD	SFO_R	-0.76	0.14	0.22	2.0e-06	3.9e-04	0.09	-0.61
120	V VOR	RD	SLF_R	-0.69	0.16	0.18	7.4e-05	3.7e-03	-0.16	-0.61
121	V VOR	V_{In}	ACR_L	0.82	0.19	0.26	7.9e-05	4.0e-03	-0.11	0.59
122	V VOR	V_{In}	ACR_R	0.93	0.22	0.31	6.8e-05	3.5e-03	-0.15	0.57
123	V VOR	V_{In}	CgC_L	0.82	0.25	0.17	2.1e-03	4.7e-02	0.12	0.47
124	V VOR	V_{In}	GCC	0.75	0.17	0.24	5.6e-05	3.1e-03	-0.13	0.64
125	V VOR	V_{In}	PCR_L	0.81	0.24	0.18	1.4e-03	3.6e-02	-0.06	0.45

Abbreviations: GCC, Genu of corpus callosum; BCC, Body of corpus callosum; SCC, Splenium of corpus callosum; ALIC, Anterior limb of internal capsule; PLIC, Posterior limb of internal capsule; RLIC, ; Retrolenticular internal capsule; ACR, Anterior corona radiata; SCR, Superior corona radiata; PCR, Posterior corona radiata; PTR, Posterior thalamic radiation; SS (IFO/ILF), Sagittal stratum (Inferior fronto-occipital fasciculus / inferior longitudinal fasciculus); EC, External capsule; CgC, Cingulum (cingulate gyrus); SLF, Superior longitudinal fasciculus; SFO, Superior fronto-occipital fasciculus; UNC, Uncinate fasciculus; FX, Fornix; ST-FX, Stria Terminalis-Fornix; _R, right-sided; _L, left-sided. PCVD, post-concussive vestibular dysfunction; DHI, dizziness handicap scale; VVAS, visual vertigo analog scale; VOMS, vestibular/ocular motor screening; NPC, near-point convergence; VOR, vestibular-ocular reflex; VMS, visual motion sensitivity; H, horizontal; V, vertical. Multiple comparisons of robust regressions adjusted for using the false-discovery rate (FDR). FDR-adjusted P values are shown in the `q.value` column. Spearman correlation coefficients between standardized diffusion and clinical measures for the control and PCVD groups are included for descriptive purposes.

Supplemental Table 1 Results of Robust Regression between Subjective Vestibular Clinical Measures (DHI, VVAS & VOMS) and Diffusion Measures

	Clinical Measure	Diffusion Metric	ROI	Robust Regression					Spearman Correlation Coefficient (ρ)	
				β	std. error	Adj. R^2	P $value_{unadj.}$	q.value	Ctrl	PCVD
126	V VOR	V_{In}	RLIC_L	0.78	0.16	0.24	1.5e-05	1.2e-03	0.01	0.72
127	V VOR	V_{In}	SCR_L	0.85	0.25	0.2	1.1e-03	2.9e-02	-0.08	0.54
128	V VOR	V_{In}	SFO_L	1.18	0.35	0.35	1.6e-03	3.8e-02	-0.23	0.63
129	V VOR	V_{In}	SLF_R	0.72	0.2	0.17	6.1e-04	1.9e-02	0.18	0.52
130	V VOR	V_{In}	SS_L	0.75	0.15	0.22	4.9e-06	5.6e-04	0.1	0.63
131	V VOR	V_{In}	SS_R	0.72	0.16	0.2	4.1e-05	2.4e-03	-0.08	0.6
132	V VOR	ODI_S	SS_L	-0.75	0.23	0.17	1.5e-03	3.7e-02	0.22	-0.56
133	VMS	FA	EC_L	0.85	0.19	0.38	5.6e-05	3.0e-03	0.09	0.69
134	VMS	FA	EC_R	0.81	0.22	0.36	4.4e-04	1.5e-02	0.08	0.68
135	VMS	FA	SS_L	0.65	0.17	0.27	3.4e-04	1.2e-02	0.32	0.72
136	VMS	MD	ACR_L	-0.78	0.21	0.28	5.4e-04	1.7e-02	-0.05	-0.62
137	VMS	MD	ACR_R	-0.81	0.22	0.3	5.2e-04	1.7e-02	-0.05	-0.63
138	VMS	MD	EC_L	-0.89	0.19	0.35	1.6e-05	1.2e-03	0.07	-0.65
139	VMS	MD	RLIC_L	-0.87	0.19	0.38	2.6e-05	1.8e-03	-0.07	-0.74
140	VMS	MD	RLIC_R	-1.03	0.24	0.42	6.3e-05	3.3e-03	-0.14	-0.7
141	VMS	MD	SS_R	-0.79	0.24	0.28	1.4e-03	3.6e-02	-0.24	-0.64
142	VMS	MD	UNC_L	-0.75	0.19	0.31	2.5e-04	9.7e-03	-0.02	-0.62
143	VMS	MD	UNC_R	-0.74	0.22	0.29	1.1e-03	3.0e-02	-0.04	-0.65
144	VMS	RD	EC_L	-0.9	0.17	0.46	4.1e-06	5.5e-04	-0.1	-0.68
145	VMS	RD	EC_R	-0.9	0.19	0.45	2.3e-05	1.6e-03	-0.01	-0.64
146	VMS	RD	FX-ST_R	-0.62	0.19	0.17	1.7e-03	4.0e-02	-0.11	-0.51
147	VMS	RD	SS_L	-0.62	0.18	0.24	1.5e-03	3.7e-02	-0.32	-0.64
148	VMS	RD	UNC_L	-0.67	0.21	0.22	2.2e-03	4.8e-02	-0.1	-0.62
149	VMS	V_{In}	ACR_R	0.81	0.23	0.28	9.9e-04	2.7e-02	0.14	0.59
150	VMS	V_{In}	EC_L	0.87	0.17	0.35	3.5e-06	5.2e-04	0.02	0.62
151	VMS	V_{In}	EC_R	0.87	0.24	0.34	5.8e-04	1.9e-02	-0.12	0.6
152	VMS	V_{In}	RLIC_L	0.77	0.21	0.29	5.8e-04	1.8e-02	0.12	0.73
153	VMS	V_{In}	RLIC_R	0.86	0.24	0.3	8.1e-04	2.4e-02	0.2	0.63
154	VMS	V_{In}	SCR_L	0.87	0.26	0.26	1.5e-03	3.8e-02	0.11	0.59
155	VMS	V_{In}	SS_L	0.79	0.19	0.32	1.0e-04	4.8e-03	0.23	0.63
156	VMS	V_{In}	SS_R	0.75	0.21	0.29	6.5e-04	2.0e-02	0.21	0.69
157	VMS	V_{In}	UNC_L	0.67	0.18	0.25	4.4e-04	1.5e-02	0.08	0.58
158	VMS	ODI_T	EC_L	-0.73	0.19	0.22	2.6e-04	1.0e-02	-0.14	-0.53
159	VMS	ODI_T	SS_L	-0.76	0.23	0.22	2.0e-03	4.5e-02	-0.25	-0.56
160	VMS	ODI_P	EC_L	-0.85	0.22	0.36	2.5e-04	9.6e-03	-0.15	-0.63

Abbreviations: GCC, Genu of corpus callosum; BCC, Body of corpus callosum; SCC, Splenium of corpus callosum; ALIC, Anterior limb of internal capsule; PLIC, Posterior limb of internal capsule; RLIC, ; Retrolenticular internal capsule; ACR, Anterior corona radiata; SCR, Superior corona radiata; PCR, Posterior corona radiata; PTR, Posterior thalamic radiation; SS (IFO/ILF), Sagittal stratum (Inferior fronto-occipital fasciculus / inferior longitudinal fasciculus); EC, External capsule; CgC, Cingulum (cingulate gyrus); SLF, Superior longitudinal fasciculus; SFO, Superior fronto-occipital fasciculus; UNC, Uncinate fasciculus; FX, Fornix; ST-FX, Stria Terminalis-Fornix; _R, right-sided; _L, left-sided. PCVD, post-concussive vestibular dysfunction; DHI, dizziness handicap scale; VVAS, visual vertigo analog scale; VOMS, vestibular/ocular motor screening; NPC, near-point convergence; VOR, vestibular-ocular reflex; VMS, visual motion sensitivity; H, horizontal; V, vertical. Multiple comparisons of robust regressions adjusted for using the false-discovery rate (FDR). FDR-adjusted P values are shown in the `q.value` column. Spearman correlation coefficients between standardized diffusion and clinical measures for the control and PCVD groups are included for descriptive purposes.

Supplemental Table 2 Results of Robust Regression between Non-Vestibular Clinical Measures (BDI, BDA & PCSS) and Diffusion Measures

				**Robust Regression**					**Spearman Correlation Coefficient (ρ)**	
	**Clinical Measure**	**Diffusion Metric**	**ROI**	β	**std. error**	**Adj. R^2**	**P value$_{unadj.}$**	**q.value**	**Ctrl**	**PCVD**
1	BAI	ODI_S	SS_L	0.93	0.23	0.13	2.2e-04	3.9e-02	-0.23	0.42
2	PCSS	MD	RLIC_L	-0.7	0.13	0.41	3.9e-06	3.7e-03	0.37	-0.73
3	PCSS	MD	RLIC_R	-0.81	0.17	0.44	1.9e-05	9.3e-03	0.47	-0.74
4	PCSS	MD	SLF_R	-0.62	0.16	0.26	3.6e-04	4.8e-02	0.46	-0.59

Abbreviations: GCC, Genu of corpus callosum; BCC, Body of corpus callosum; SCC, Splenium of corpus callosum; ALIC, Anterior limb of internal capsule; PLIC, Posterior limb of internal capsule; RLIC, ; Retrolenticular internal capsule; ACR, Anterior corona radiata; SCR, Superior corona radiata; PCR, Posterior corona radiata; PTR, Posterior thalamic radiation; SS (IFO/ILF), Sagittal stratum (Inferior fronto-occipital fasciculus / inferior longitudinal fasciculus); EC, External capsule; CgC, Cingulum (cingulate gyrus); SLF, Superior longitudinal fasciculus; SFO, Superior fronto-occipital fasciculus; UNC, Uncinate fasciculus; FX, Fornix; ST-FX, Stria Terminalis-Fornix; _R, right-sided; _L, left-sided; BAI, Beck anxiety inventory; BDI, Beck depression inventory; PCSS, post-concussion symptom scale; PCVD, post-concussive vestibular dysfunction; Multiple comparisons of robust regressions adjusted for using the false-discovery rate (FDR). FDR-adjusted P values are shown in the `q.value` column. Spearman correlation coefficients between standardized diffusion and clinical measures for the control and PCVD groups are included for descriptive purposes.
